# A topological characterization of flooding impacts on the Zurich road network

**DOI:** 10.1371/journal.pone.0220338

**Published:** 2019-07-31

**Authors:** Ylenia Casali, Hans R. Heinimann

**Affiliations:** 1 Future Resilient Systems at the Singapore-ETH Centre (SEC), ETH Zurich, Singapore; 2 Department of Environmental Systems Science, ETH Zurich, Zurich, Switzerland; Tel Aviv University, ISRAEL

## Abstract

Infrastructure systems are the structural backbone of cities, facilitating the flow of essential services. Because those systems can be disrupted by natural hazards, risk management has been the prevailing approach for assessing the consequences and expected level of damage. Although this may be a valuable metric, the practice of risk assessment does not represent how hazards affect a network of assets on a larger scale. In contrast, network topology metrics are useful because they evaluate the performance of network infrastructures by looking at the system as a whole. As described here, we began this study to improve our understanding of how flooding events affect the topological properties of road networks, in this case, the urban road infrastructure of Zurich, Switzerland. Using maps of flooding risk, we developed a procedure to extract the damaged networks and analyze the centrality metrics for peak water levels on the surface of the city. Our approach modelled roads as edges and junctions between roads as nodes. The betweenness centrality metric characterizes the importance of nodes or edges for any type of exchange within a network, whereas the closeness centrality metric measures the accessibility of a specific node to all the other nodes. This investigation produced three main findings. First, descriptive analyses showed that the characteristics and patterns of nodes and edges changed under the flooding events. Second, the distribution function of centrality metrics became heavier in the tails as the flood magnitude increased. Third, the associated strain shifted critical nodes to areas in which those nodes would not be important under normal conditions. These findings are essential for identifying crucial locations and devising plans to address risks. Future projects could expand our approach by including traffic flow to move the analysis closer to real-world flows, and by studying the accessibility under emergency conditions at local levels.

## Introduction

Along with clustering and the aggregation of assets in space, urbanization has been a driving force of global change. Within those clusters, the values at risk are continually increasing and–when exposed to patterns of natural hazards–have resulted in a tremendous rise in the costs associated with expected damage. Risk assessment is a method used to analyze the consequences to single assets or small sets of assets that are exposed to the pattern of a specific hazard. However, that approach does not take into account that assets are connected and that a triggering hazardous event can cause such damage to spread in a cascading manner through an entire network. Therefore, the question becomes how does one characterize the response of that network of assets when subjected to natural hazards. Network science views real systems as being formed by interacting parts that represent infrastructures. Information about their geographical locations and the relationships among roads is used to detect the robustness of roads. However, little is known about how natural hazards affect the topological properties of a network. The scientific literature has presented three research streams for addressing the response of real road networks to alterations in their topological structure. The first method examines scalar changes in centrality metrics to determine patterns of variation. Such research began during the second half of the 20^th^ Century, within a social systems context. For example, Bavelas [1] and Beauchamp [2] developed early studies in closeness centrality metrics to identify the group of nodes that enable efficient communication of information. Freeman [3] proposed the betweenness centrality (BC) metric to measure the location at which information flow is controlled within a network. Strano et al. [4] showed that the growth of the road network in a suburban area north of Milan is governed by two processes that they measured quantitatively by studying differences in the edge BC averages. They also quantified the impact of single roads in the growth process. Pregnolato et al. [5] compared various strategies for adapting to flooding events along the road network of Newcastle upon Tyne by evaluating changes in the average and maximum values of node BC. A second stream of research has focused primarily on centrality distributions of the whole set of nodes and edges for a network. For example, Barrat et al. [6] have investigated the effect of spatial constraints on BC distribution and found that, when those constraints become important, then BC displays larger fluctuations. Two factors influence such fluctuations: 1) the presence of locations that connect geographically distant regions and 2) the relative position of the barycenter in the geographical space. Since that report, the distributions of BC metrics have been characterized by researchers such as Lämmer et al. [7], who analyzed the 20 largest German cities and determined that the BC metrics follow power law distributions. Likewise, Crucitti et al. [8] studied the distributions of centrality metrics for 18 cities worldwide and learned that BC follows an exponential distribution in self-organized cities but a Gaussian distribution in planned cities. More recently, Kirkley et al. [9] have examined a dataset of 97 cities across the world, and have observed that BC is distributed as truncated power laws, with some invariant properties that make a network robust to major alterations, including changes in its topology and edge weight structure. A third stream of research has used spatial patterns of centrality metrics to identify the main changes in a particular topology. Crucitti et al. [10] have discussed in detail the spatial distribution of centralities in a planned city versus a self-organized city. An investigation by Barthélemy et al. [11] of modifications to the topology of the Paris road network over time has revealed different periods in which important reorganizations of the spatial distribution of centrality have corresponded to specific interventions. Kermanshah and Derrible [12] have studied the robustness of road networks in response to extreme flooding events in New York and Chicago. They have used BC metrics to measure changes in roads before and after those events by looking at the maximum and average values and at the spatial locations of edge BC. Applying that approach has enabled them to observe a shift in the concentration of roads in parallel with higher edge betweenness centralities.

Despite all of these advances, a coherent means is still lacking for characterizing changes in network properties under major disruptions. Taking up this challenge, we designed this present study to obtain a logical methodology for comparing the pre- versus post-event conditions of large-scale road networks. Our investigation included a descriptive analysis of centrality metrics and assessed the variability of those metrics as well as the spatial arrangement of critical network components. Out of necessity this project involved only a single geographical area, i.e., the city of Zurich, and focused only on flooding events.

## Methodology

### 2.1 Study area

Our study area was the city of Zurich, in northern Switzerland. This city represents an urban region, as delimited by administrative boundaries set in 1934. Its 12 districts cover approximately 88 Km^2^, with a population of approximately 415,682 in 2016 [13]. The city is located at the northern tip of Lake Zurich. Here, the Limmat River flows northeastward, dividing the urban landscape into two parts. A second river, the Sihl, flows northward from the southern border in the western part of Zurich until it joins the Limmat River in the central area. Extreme floods have been recorded for several centuries. The most recent, caused by heavy rain, occurred in summer 2005, prompting the installation of flood protection projects by Kanton Zurich [14].

### 2.2 Data

We relied upon road data for Zurich from the Swiss Federal Office of Topography (Swisstopo), as well as flood hazard maps and digital elevation models (DEMs). For the road information, we used the swissTLM3D 1.1 data model for Year 2012 [15]. This dataset represents roads as line features in a vector shapefile format, projected in the Swiss CH1903 coordinate system. The flood hazard maps, provided by TK consult AG, were derived via a hydrological model calibrated according to the historical flood of 2005. That model simulated the affected areas corresponding to several flood events associated with different water discharges at the Lake Zurich inlet, Sihl River, and Limmat River. It included the perimeter of buildings as a no-flow-boundary condition, which meant that the flux of water throughout the perimeters was null. For our evaluation, we used flood events that covered a 100-year return period and a 300-year return period. From the available hazard maps [16], we selected those that displayed the spatial distribution of flooded areas in Zurich. Those data were polygons featured in a vector shapefile format. The DEMs were obtained from the Office for Spatial Development of Kanton Zurich. For this, we used the digital terrain model (DTM) and the digital surface model (DSM). The former represents the elevation of a ground surface without any objects while the latter represents the elevation of the earth’s surface, including the objects on it. Here the dot matrix was 50 cm and included accuracies of 20 cm in location and 10 cm in height.

### 2.3 Analytical framework

After pre-processing to convert geographical data into a network representation, we analyzed the processed networks with centrality metrics. Distributions were examined with ArcMap 10.4 software on the ArcGIS environment for the geographical analyses, the Arcpy and Igraph packages in Python for topological analysis, and R packages for the statistical analysis.

#### 2.3.1 Data pre-processing

A network is defined as a set of points in a space connected by a set of edges that are links between node pairs. Modelling of network representations is commonly used to study systems having different natures. Here, we designated edges as roads and nodes as road junctions. As the primal representation, it is the opposite of a dual representation, where nodes would be roads, and road junctions, edges. The entity of a road can be examined from different perspectives [17–18]. Our study specified a road as the contiguous space between two separated crossroads in the geographic space. In our dataset, a single road entity was divided into multiple segments. This necessitated first pre-processing the data by using the unsplit line tool in ArcGIS. For building the network, we needed to identify the node and edge features from the geographical data. To do so, we used the network topology toolbox in ArcGIS to create two layers corresponding to the node and edge features from the road vector data. Within a layer, each node or edge was given a distinct ID number. For developing the topological analysis, we created an edge list of the road network by developing a script that read the ID number of every single node and then assigned the respective node-pair IDs to each edge. In this way, we modelled a network comprising the entire set of roads for Zurich. To generate a network representing roads not inundated by flood waters, we first selected the edges that intersected those flooded areas and then erased them from the original network so that only roads unobstructed by water were retained. In 2012, bridges and tunnels accounted for no more than 2% of the total number of roads, which meant that the network was almost on the same plane. However, because most of the flooded areas were in close proximity to rivers, where the highest density of bridges were also located, we had to determine which of those bridges were actually inundated. First, we calculated the height of each bridge by applying both the DTM and DSM. Second, if the bridge was taller than the depth of the water, it was not deleted from the original network. In risk analysis, a common approach is to look at the worst-case scenario in order to account for the largest loss that may occur during an event. Here, we selected the maximum peak scenario of the flood model to evaluate the flooded network during the peak scenarios for the 100-year and 300-year return periods, and then extracted the edge lists for these networks.

Planners classify road types according to their widths, which characterize their physical form [17]. Traffic flows along lanes that are the part of a road cross section set aside for one-way movement of vehicle streams. In Switzerland, lane widths typically range from 3.45 to 4.00 m for urban arterials and freeways and from 3.00 to 3.65 m for minor or local roads [19]. For studying the system accessible by vehicles only, we had to select roads that, structurally, were meant to supply vehicle flow. The original Zurich road data provided information about road classes and width ranges [15]. In the ArcGIS environment, we assigned to each edge segment the relative minimum width of the associated road. We then selected roads at least 3 m wide. That set of roads formed the network that would be easily accessible for traffic. We extracted the networks covering the peak scenarios for 100-year and 300-year return period events. When we analyzed the network with roads of that size (≥3 m), their widths were used as weights for estimating BC, a component that required an additional analytical layer to distinguish among road types. The traffic flow volume is equal to the density times speed, and density indicates the number of vehicles per unit length. By assuming that the speed remains approximately constant within a city, the flow volume is observed to be a function of density, dependent on the number of lanes. Based on this logic, we considered the road width as a proxy of flow density, which enhances the applicability of our analysis to real-world flows.

#### 2.3.2 Centrality metrics

For this project, we developed three types of centrality analysis. In general, nodes and edges are the basic components of a network, and network topology is defined by the relationships between the total number of nodes *N* and edges *E*. Centrality metrics characterize the importance of nodes or edges to a network. Here, we studied the node BC, edge BC, and closeness centrality. Usually, many paths exist between two nodes. The shortest path is the one between a certain pair of nodes such that the total sum of the edge weights is minimal. In an unweighted network, each edge is treated the same. The BC metrics adapted to a traffic-flow-oriented betweenness centrality metric by weighing the shortest paths based on the road width. Centrality values depend on the size of networks and the numbers of nodes and edges. Therefore, we used normalized centrality metrics to compare the centrality results from the baseline conditions and our two flooding scenarios. We employed the Igraph package to evaluate those metrics on Python.

We defined normalized node betweenness centrality as (1):

$$NBC(i)=\frac{1}{\left(N-1)(N-2\right)}\sum_{s\neq i\neq t}\frac{\sigma_{st}(i)}{\sigma_{st}}$$ (1)

where *σ*_*st*_ is the number of shortest paths going from a source node *s* to a target node *t*, σ_st_(i) is the number of shortest paths going from node *s* to node *t* that pass through node *i*, and *N* is the total number of nodes. In an undirected network, the maximum number of node pairs would be (*N* − 1)/2. Because node *i* cannot be an extreme of the shortest paths, NBC is normalized by the maximum number of possible pairs of nodes that becomes (*N* − 1)(*N* − 2)/2 [3,10]. Based on this definition, nodes at the endpoints have centrality values equal to zero, which is also the minimum because those values can never be negative. This metric characterizes the importance of the node *i* in the organization of flows in the network [20].

Normalized edge betweenness centrality is defined as (2):

$$EBC(e)=\frac{1}{N(N-1)}\sum_{s\neq t}\frac{\sigma_{st}(e)}{\sigma_{st}}$$ (2)

where σ_st_(e) is the number of shortest paths going from node *s* to node *t* that pass through edge *e*. Although EBC evaluates the same pattern as NBC, its reference is the edge feature instead of the node features. This metric is normalized to its maximum number of node pairs (*N* − 1), for a totally connected graph having *N*(*N* − 1)/2 edges.

We calculated the normalized closeness centrality per (3):

$$CC(i)=\frac{N-1}{\sum_{i\neq j}l_{ij}}$$ (3)

where *l*_*ij*_ is the shortest-path length between node *i* and node *j*. As such, that variable is the smallest distance that separates node *i* from all other nodes in the network. The metric is normalized to the number of node pairs (*N* − 1) having node *i* as one extreme. It measures the extent to which a particular node *i* is near all other nodes along those shortest paths [10].

#### 2.3.3 Descriptive analysis

Our descriptive analysis characterized the changes in the numbers of nodes and edges and in the centrality values from baseline conditions to the two flooding events. During those events, the closure of a certain set of roads may have altered the centrality values at a single node/edge location. We identified four classes of changes: 1) node or edge flooded and, therefore, closed in the system under a flood scenario; 2) increase in the centrality value at a single node or edge from the baseline; 3) decrease in that value; or 4) no impact from flooding, so that the centrality value did not change from the baseline level. We calculated the differences between normalized centrality values under baseline conditions as well as in the flooding scenario, examining the entire network system (total number of roads in Zurich) because we wanted to investigate the overall trend among centrality values.

#### 2.3.4 Parametric characterization of the BC distribution

The distribution functions are used to systematically characterize the variability of the variable of interest, which, in this case, is the betweenness centrality. If we are interested in the tail of the distribution, which has been traditionally the case for risk management, we have to use specific models for accurately representing this tail. The extreme value distributions are used in a traditional risk management approach, whereas the power law distributions are popular in the field of complexity science. Power law distributions are defined as *p*(*x*) ~ *x*^−*α*^ (4) when *x* > *x*_*min*_. Although the power laws cannot characterize the “left-hand” part of the distribution, they constitute a straightforward approach to characterize the upper tail using two parameters, the exponent alpha and the lower bound *x*_*min*_ at which the power law distribution has to be cut off. The Kolmogorov–Smirnov (KS) statistic is a goodness-of-fit measure used in power law analyses [21]. We used the powerlaw package in Python for conducting the analyses.

#### 2.3.5 Spatial presentation of results

The spatial analysis was conducted to visualize the centrality results on maps. After importing the results from the centrality calculations of Python on the ArcGIS environment, we joined those results with the node and edge layers so that each node/edge was associated with its centrality value. This produced shapefiles relative to each centrality value, which we could then display, as quantitative results, on maps or shapefiles that corresponded to actual geographical locations. For our purposes, high centrality values were critical because they referred to the most important nodes/edges. To identify their particular locations, we selected the 0.99-quantiles from the centrality results and presented only those on the map of Zurich.

## Results

After obtaining a descriptive characterization of network properties, we analyzed the statistical distributions of the centrality metrics to understand their variability in response to flooding events. Finally, we investigated the spatial distributions of the centrality results.

### 3.1 Descriptive analysis

The descriptive analysis yielded node and edge characteristics, patterns for the two flooding scenarios (100 and 300 years), and the three centrality metrics. Although flooding considerably altered the topological properties of the network, a small percentage of nodes and edges could not be characterized by those scenarios. The overall flow capacity by the network was represented by betweenness centrality metrics that, when compared with baseline conditions, showed changes mostly at the nodes/edges. The baseline conditions included 6704 nodes and 9931 edges. The number of nodes decreased to 6676 (decline of 0.4%) for the 100-year flood and to 6472 (–3%) for the 300-year event. Meanwhile, the number of edges decreased to 9796 (–1%) for the 100-year peak and to 9381 (–6%) when compared with the baseline level. These results indicated that the flooded nodes and edges represented a small part of the network, and that major disruptions emerged during the 300-year peak event. Centrality metrics characterize the importance of nodes and edges for overall flow capacity. The transition from ‘normal’ to ‘flooded’ conditions results in one of four responses by single nodes/edges. As shown in Table 1, values increased for approximately 40% of the nodes but decreased for approximately 45% when NBC was considered. For EBC, values were increased for approximately 46% of the edges but decreased for 50% of them. These results demonstrated that, overall, patterns were altered for node betweenness and edge betweenness centralities. In contrast, an examination of closeness centrality indicated that values were decreased for approximately 96% of the nodes. Overall, the analyses of edge and node betweenness centralities revealed more heterogeneous changes than did the closeness centrality.

**Table 1:** Changes in the number of nodes from the peak scenario for normalized node betweenness centrality and closeness centrality, and changes in the number of edges for the normalized edges betweenness centrality.

Node-Edge Transition					
	*Return Period*	*Increase*	*Decrease*	*No change*	*Flooded*
*Node Betweenness Centrality*	100	2933 (44%)	2886 (43%)	857 (13%)	28 (0.4%)
	300	2453 (37%)	3183 (48%)	836 (12%)	232 (3.0%)
*Edge Betweenness Centrality*	100	4704 (47%)	5091 (51%)	1 (0%)	135 (1%)
	300	4496 (45%)	4885 (49%)	0 (0%)	550 (6%)
*Closeness Centrality*	100	75 (1%)	6601 (98%)	0	28 (0.4%)
	300	75 (1%)	6397 (95%)	0	232 (3.0%)

Four causes of change in nodes/edges were possible: 1) increase in centrality values, 2) decrease in values, 3) values maintained at the same levels, or 4) exclusion from the network because those nodes/edges were flooded. Changes were calculated as the differences between normalized values in a flood scenario and those under baseline conditions. Percentages indicate the ratio of the number of nodes (edges) to the total number of nodes (edges) when compared with baseline conditions, i.e., 6704 nodes and 9931 edges.

### 3.2 Analysis of centrality distribution function

The empirical distribution functions of the centrality metrics for our three scenarios differed significantly in their tails. For the BC results, this demonstrated an increase in the heaviness of the tail at higher flood magnitudes. A heavier tail meant that either the significance of critical edges needed to maintain greater network performance was enhanced or the failure of some particular roads had a larger impact on the network. When we considered systems composed only of roads at least 3 m wide, we found that the 100-year flood distribution was greater than that of the 300-year flood. Our closeness centrality results also showed a decline in distribution values.

From a baseline of 4795 nodes and 6470 edges, the scenario featuring a 100-year flood event showed a decrease in node and edge numbers to 4773 (drop of 0.5%) and 6396 (drop of 1.1%), respectively, whereas the 300-year flood resulted in respective reductions in node and edge numbers (4572, down by 5%; and 5999, drop of 7%) when compared with baseline conditions. Here, we calculated NBC and EBC values by using the width capacities as network weights. Basic statistical analyses of the normalized centralities values showed that, under baseline conditions, the maximum values were 0.08 for the node betweenness centrality and 0.07 for the edge betweenness centrality. For both metrics, the maximum values were 0.12 for the 100-year scenario and 0.13 for the 300-year scenario. The standard deviation of NBC was approximately 8.4·10^−3^ for the baseline, 9.7·10^−3^ for the 300-year flood, and 1.3·10^−2^ for the 100-year scenario. For EBC, the standard deviation was approximately 6.8·10^−3^ for the baseline, 8.1·10^−3^ for the 300-year flood, and 1.1·10^−2^ for the 100-year scenario. For the node-BC, the 0.5-quantiles were approximately 9.4·10^−4^ for the baseline and it decreased to 8·10^−5^ in the 100-year flood, while in the 300-year flood to 4.4·10^−5^. For the edge-BC, the 0.5-quantiles were approximately 6.8·10^−4^ under baseline conditions, but those decreased to approximately 6·10^−4^ for the 100-year flood and to 3.6·10^−5^ for the 300-year flood. While the 0.99-quantile of node betweenness centrality was around 4.3·10^−2^ in the baseline, and it increased to 5.6·10^−2^ in the 300-year scenario and to 7.1·10^−2^ in the 100-year scenario. While, when we compared the 0.99-quantiles of edge betweenness centrality, it was 3.4·10^−2^ in the baseline, 4.7·10^−2^ in the 300-year scenario and to 6.5·10^−2^ in the 100-year scenario. These results indicated that the values of the distributions increased in variability as flooding intensified, and that they became more broadly distributed during the 100-year flood.

**Fig 1:**
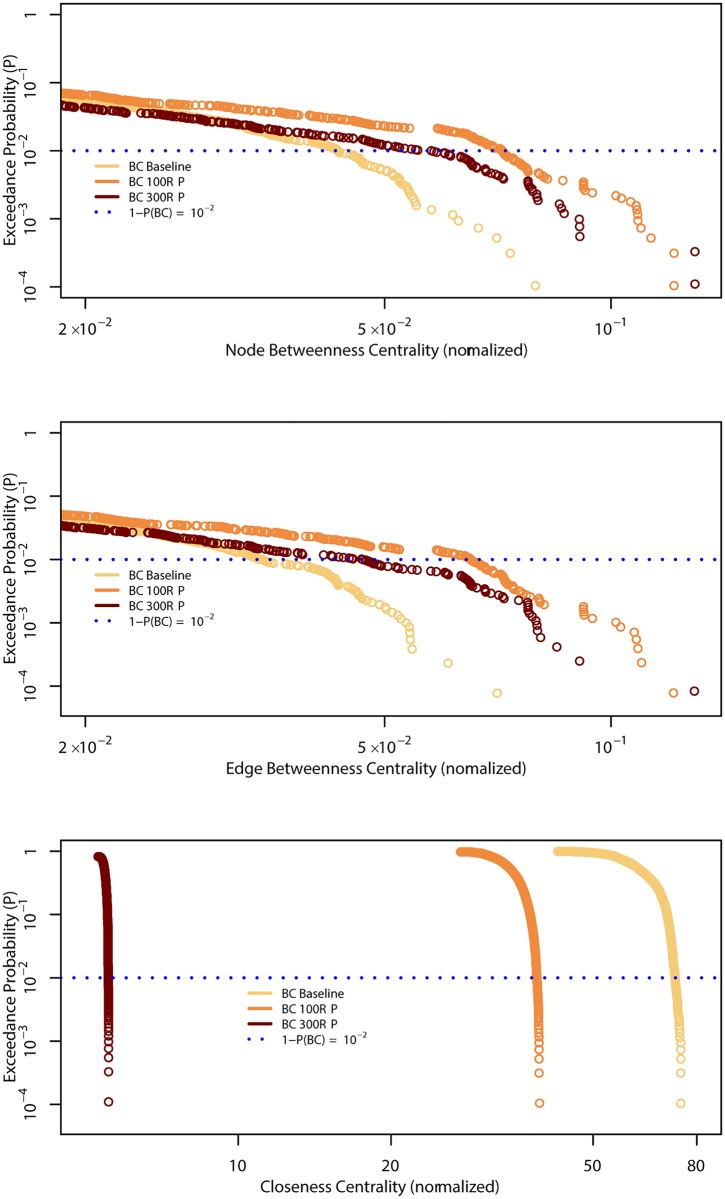
Complementary distribution functions for baseline conditions and 2 flooding scenarios, based on main roads at least 3 m wide. Edge and node betweenness centralities were calculated using average road widths as network weights.

The results of the normalized closeness betweenness centrality showed that the maximum values and the standard deviation decreased from the baseline condition to the 300-year flood, respectively from around 74 to 6 for the maximum and the standard deviation from around 8 to 2. As in the previous analysis, the quantiles always decreased values from the baseline to the 300-year peak. Fig 1 illustrates the complementary distribution functions, with the exceedance probability 1−P(x) being a function of normalized centrality values. For our BC results, we displayed only the tails of the distributions because those values were critical when trying to understand the relative importance of nodes and edges closer to the upper extremes, all of which had a high incidence of shortest paths. The tails of the betweenness centralities showed that the baseline condition values had higher probabilities than the flooding scenario values for up to approximately 10^−2^. Beyond that point, a switch occurred in the baseline and 100-year curves, with the latter showing higher probabilities than the former. A second switch in the distributed values occurred at approximately 2·10^−2^, where the probabilities of the 300-year flood centrality values became higher than the probabilities of the baseline. The 100-year flood values had higher probabilities than the baseline or the 300-year flood scenarios. These findings meant that, when considering the network created to support vehicle flows, the critical locations associated with the 100-year scenario became more important than either the baseline conditions or the 300-year flood scenario, even though the degree of disruption was greater for the latter than for the 100-year flood.

The parametric characterization of the upper tail of the NBC distribution functions yielded alpha values of 3, 6.4, and 1.9 for the baseline as well as the 100-year and 300-year flood scenarios, respectively. The corresponding values for the EBC distributions were approximately 2.5, 2.2, and 1.9, which were higher than those observed in other German cities [7]. Here, NBC and EBC denoted the lowest alpha value during the 300-year flood scenario. *x*_*min*_ ranged from 0.003 to 0.06 for NBC and from 0.01 to 0.02 for EBC. The KS statistic is the maximum distance between the data distribution function and the power-law-fitted distribution. In case of NBC, the KS statistic was 0.07 in the baseline and 0.06 in the two flooding scenarios, whereas it was 0.05 in the baseline and 0.04 in the two flooding scenarios for EBC. Because the lowest KS values were observed in the flooding scenarios, BCs became closer to a power law distribution under the flood strain when compared with that in the baseline condition. We tested the hypothesis that the empirical distribution function is equal to the theoretical power law distribution function above the threshold value using the KS test. The critical distances indicated that the p-values were lower than 0.01, indicating that we should reject the hypothesis that the empirical distribution and corresponding power law distributions are obtained from the same population. The test of other distributions, exponential and lognormal, and the empirical distribution were from the same population had to be rejected, too.

### 3.3 Spatial presentation of results

In the third part of our analysis, the spatial pattern of nodes/edges with high relevance to overall network performance shifted from the baseline scenario to the two flooding scenarios.

**Fig 2:**
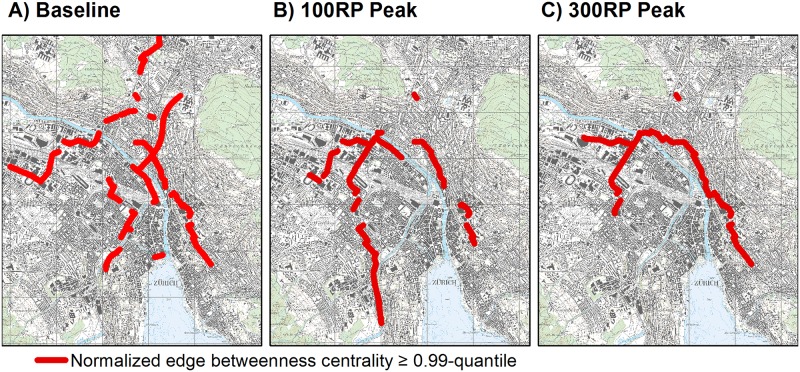
Spatial distribution of edge betweenness centrality in Zurich network of roads at least 3 m wide, under the following scenarios: Baseline conditions, peak flood within 100-year return period, or peak flood within 300-year period. For each network edge, EBC was evaluated by dividing number of shortest paths through edge by total number of shortest paths within network. Values of edge betweenness were normalized with (N)(N-1), where N was number of nodes under baseline conditions. Figure presents values equal to or larger than 0.99-quantiles. Reprinted from National Map 1:25000 on sheet 1091 under a CC BY license, with permission from the Federal Office of Topography Swisstopo (original copyright 2019).

During hazardous periods, emergency managers must identify which sequence of roads is critical for managing traffic flows inside a city. To test this, we first analyzed edge betweenness centrality and the spatial distribution of the edges at the tails, which corresponded to edges having normalized BC values equal to or higher than the 0.99-quantiles. We also examined how the pattern of critical edges changed for roads wider than 3 m. As shown in Fig 2, under baseline conditions, the critical edges were located mostly in the central area and then moved away from the city center in four directions: northwestward, southwestward along the Sihl River, southeastward, and northeastward along connections with the A1-east national freeway. Most of those critical edges occurred on narrow paths, such as entrances to bridges and tunnels that crossed rivers and railway lines. Edges with high centrality developed toward the accesses to the A1-east and A3/A4 national freeways, located in the northwestern and southwestern parts of Zurich. However, those spatial distributions were altered in the flooding scenarios. For the 100-year return period peak scenario (Fig 2B), some critical edges shifted locations, especially in the northern and central areas. A new path direction also emerged from the Limmat side toward the southwestern part of the city. For the 300-year scenario (Fig 2C), those critical edges differed from the 100-year scenario because they did not extend westward into the southern part of Zurich. Under the 300-year scenario, the network featured a small density of roads in the southwestern area. Therefore, any closures of connections in that direction would have reduced the number of shortest paths for travel to that area. This also demonstrated that it was possible to identify critical edges in an infrastructure that could still support a significant passage of shortest paths and, consequently, of flows.

**Fig 3:**
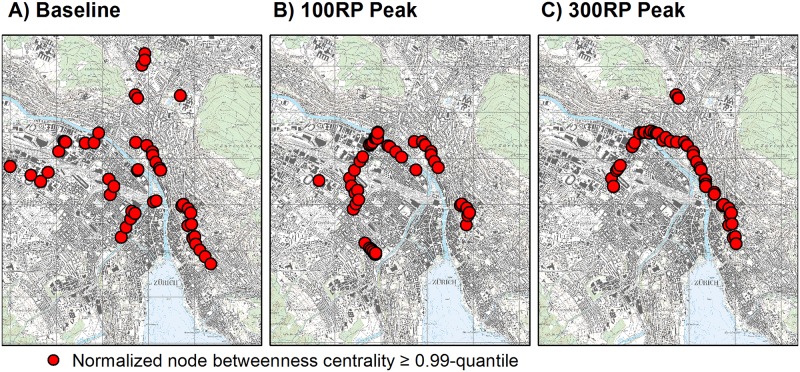
Spatial distribution of node betweenness centrality for Zurich road network under baseline conditions (A) or peak flood scenarios (B, C). Results were calculated for system of roads at least 3 m wide. Betweenness centrality was evaluated for each network node by dividing number of shortest paths through nodes by total number of shortest paths within network. Values of betweenness were normalized with (N-1)(N-2), where N was number of nodes under baseline conditions. Figure presents values equal to or larger than 0.99-quantiles. Reprinted from National Map 1:25000 on sheet 1091 under a CC BY license, with permission from the Federal Office of Topography Swisstopo (original copyright 2019).

As the second step in this test, we studied the spatial pattern of normalized NBC results, selecting nodes with values equal to or above the 0.99-quantiles, which represented the critical junctions between roads (Fig 3). Here, values were similar between NBC and EBC. Under baseline conditions, critical nodes were mostly spread in four directions from the center of Zurich. In contrast, flooding events were associated with critical nodes that were narrower on the paths. In particular, the flooding scenarios generally produced two paths of critical nodes: 1) from the central area toward the Limmat River, with further development south of that river, in the western part of Zurich; or 2) path development southwestwardly from the central area. These patterns confirmed our findings for EBC and showed that, for the 100-year flood scenario, high centrality values extended more to the southwest when compared with the more westward movement detected under the 300-year scenario.

**Fig 4:**
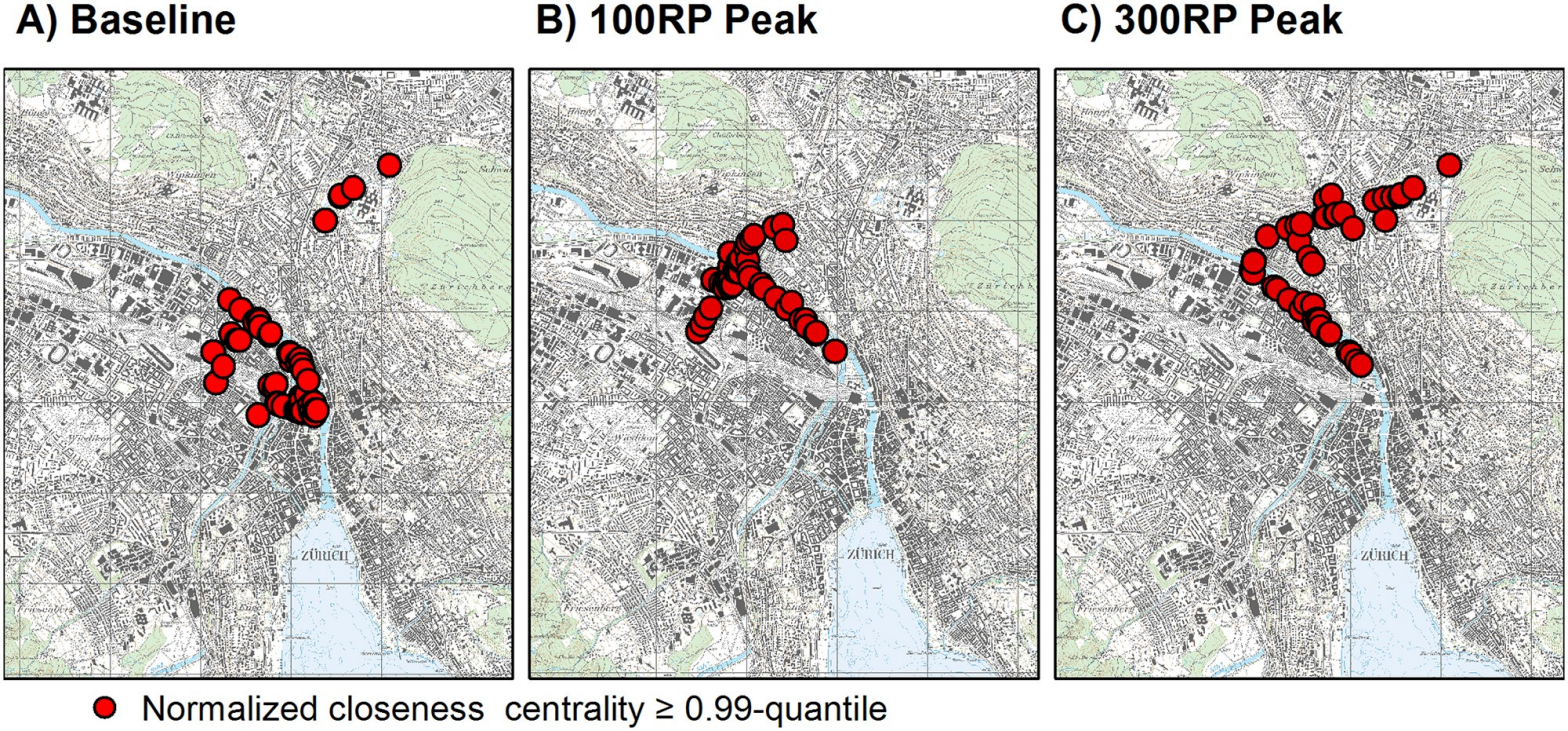
Spatial distribution of normalized closeness centrality for network featuring roads at least 3 m wide. Values for CC were calculated for each node, dividing by total sum of shortest-path lengths within network, and were normalized with (N-1), where N is number of nodes for given scenario. Figure presents values equal to or larger than 0.99-quantiles. Reprinted from National Map 1:25000 on sheet 1091 under a CC BY license, with permission from the Federal Office of Topography Swisstopo (original copyright 2019).

Finally, we considered the spatial distributions for CC (Fig 4). Under baseline conditions, the selected high-value nodes were mostly clustered in the city center, near the central railway station where the two rivers join. Other nodes were identified along the Limmat River and in the northeastern area for infrastructure that connects Zurich with the A1-east national freeway. Under the flooding scenarios, those nodes shifted to the northern areas close to the Limmat River as well as toward the connections with the A1-east national freeway. This spread of nodes toward those freeway connections was more extensive under the 300-year scenario (Fig 4C).

## Discussion and conclusions

We used descriptive analysis, variability of the metrics, and spatial distributions on the entire road system structure of Zurich to determine how centrality metrics might be altered by an historic flooding event. Both the characteristics and patterns of nodes and edges changed in response to 100- and 300-year events. Here, the betweenness centrality metric characterized the importance of exchange within a network, whereas the closeness centrality measured the accessibility. However, when compared with the total number of nodes and edges existing under baseline (pre-flood) conditions, only a small percentage of them were affected due to the unique geography associated with this city. Flooding was restricted to areas near water bodies in the central and western areas—in particular, the alluvial fan of the Sihl River—whereas the eastern and northern areas were at higher elevations. A survey of nodes and edges indicated that changes were greater for the betweenness centrality metrics than for closeness centrality, and values for the latter mainly showed decreases. This was a result of flood-related road closures, which meant that the shortest-path lengths between node pairs could only increase from baseline conditions, and therefore, they have less influence on closeness centrality than on betweenness centralities. Most research has tended to focus on the unique influence BC can have when characterizing changes to road networks either during flooding events [12] or simply as a result of the passage of time [4,11]. This is mainly because betweenness centrality is a measure of the contribution of a link in the organization of network flows [20]. Moreover, closeness centrality results are strongly dependent on the geographical position of nodes [22], making that metric sensitive when defining the boundary of a network [10]. Our findings complement previous analyses of topological changes in road networks because they demonstrated how the trends changed for the three centrality metrics tested here.

Our analysis of BC and the empirical distribution function also showed that graphed tails grew heavier as the flood magnitude increased. This response was reflected by the rises noted for standard deviations and the 0.99-quantiles. Furthermore, the KS statistic in case of power law distributions decreased in flooding scenarios, indicating that the tails were closer to the power law under the flood strain when compared with that in the baseline condition. As such, in the flooding scenarios, nodes or edges with high BC values were relatively more important than those calculated for the baseline network. In particular, the 100-year flood showed the heaviest distribution when we looked only at roads at least 3 m wide. Furthermore, closeness centrality distributions decreased as the flood magnitude increased. The most critical BC values were those closest to the greatest extremes because they referred to the nodes and edges with the highest frequency of shortest paths passing through, i.e., locations with the most abundant city traffic. Our results were also supported by those previously reported from studies of BC distributions for datasets in other cities. For example, Lämmer et al. [7] determined that NBC follows a power law distribution for the road networks of several German cities. That research group concluded that low values for the distribution exponent could be interpreted as the shortest paths being highly concentrated at the most important intersections. Another investigation using a large world dataset showed that BC metrics could be approximated by a truncated power law [9]. In contrast, in [8] NBC followed an exponential or Gaussian distribution rather than a power law distribution. Because the KS test rejected the hypothesis that the tail of BC distribution followed a power law in our data and no lognormal or exponential distribution was revealed, we evaluated the BC basic statistics and determined that the changes in curve variability provided an essential key for understanding the effects of flooding on a real road system. Therefore, if we were going to be able to assess the response of a specific city to a certain hazard, then it would be crucial that we look at whether patterns have changed for the highest-value nodes or edges, because each of them refers to a unique position within an urban system. Our results also helped extend the discussion of Kirkley et al. [9], who examined the distributions of an entire network and demonstrated the invariance of BC distributions and, therefore, the robustness of the metric for measuring major alterations in that network.

For our third point, we conducted a spatial analysis of centrality metrics to show that the spatial pattern of centralities with values equal to or larger than the 0.99-quantiles shifted locations in the three scenarios. This meant that sites with a high impact on system performance changed their geographical positions during our test scenarios. The betweenness centrality results also proved that those sites were located on the main paths of Zurich under baseline conditions. This result confirmed those of [10], who observed that NBC captured the continuity of prominent urban routes across numerous intersections, as well as through changes in direction and focal urban spots. In particular, we noted here that narrow paths, e.g., bridges or tunnels, which crossed rivers or railway lines, tended to have higher betweenness centrality values. This was because those paths united different sections of the city that would otherwise be divided by gaps. Our results also supported observations by [10], who detected the emergence of NBC corresponding to important bridges. Those main paths changed locations during flooding events. In our flooding scenarios, a new path emerged in the western area, connecting with the Limmat River side toward the southern part of Zurich. This behavior might be explained by the closure of most roads in the west-central portion of the city but a simultaneous maintenance of links with the central Limmat River and the southern part of Zurich, so that the shortest paths passed on the western roads. Distribution of critical edges and nodes was more extensive to the south during the 100-year flood than during the 300-year flood because, under the latter scenario, fewer main roads were accessible in the west-central area. Therefore, the closures of some main roads were so devastating that some important connections collapsed. Furthermore, access became too difficult within the southern part of the city because there was not a sufficient number of shortest connecting paths. Whether one considered the network as a whole or looked only at roads meeting a certain minimum width, our closeness centrality results indicated highly clustered distributions of critical nodes in the central part of Zurich. We expected this outcome because closeness centrality generally clusters within the central part of a geographical space [22]. Crucitti et al. [10] have also uncovered a strong tendency for closeness centrality to group higher values at the center of an image. When we examined our flooded scenarios, we noted a northward shift in higher values of closeness centrality because the particular geography of Zurich makes its central roads more vulnerable. Therefore, because the city center is limited in expansion southward by the lake, that central portion can only move northward.

Our findings have implications for planners, policy-makers, and scientists. Maps of flood risk provide information about tangible losses from a hazard event, with costs commonly being quantified according to the level of economic damage or the number of people affected. Our use of topological properties is another quantitative tool that can evaluate the impacts of a hazard on the capacity for traffic to move within a road network. Here, we relied upon changes in centrality to account for how the road system as a whole was altered. Planners can use those results to rank the relative importance of individual roads or, more generally, determine which areas will be most affected. From that, policy-makers can utilize the information to derive preventative actions for minimizing the impact of future flood hazards. For scientists, our study demonstrated that centrality metrics contribute to realizing the influence a distributed hazard has on a road network. Here, the analysis of BC distribution functions over time revealed that the effect of the two flooding events on the tails was similar to that seen as a road system ages. Therefore, topological metrics alone can help us detect any shift in properties as such a system changes.

This research project had some limits–first, because we used only Zurich as our case study. Because topological properties depend upon the particular geography of a city, different results might be obtained when centrality metrics are evaluated elsewhere. Furthermore, we did not use any traffic data but instead employed only data relative to the road structure. This approach meant that we omitted any information about congestion or the most common commuter routes within Zurich.

Future work might compare the effects of flooding on the topology of road systems characterized by different urban plans or geographical constraints. Incorporating traffic information would also aid in producing a more realistic view of conditions.

Finally, follow-up investigations could be used to characterize the spatial variability of centralities, especially the closeness centrality, in limited areas of the network. This approach can be applied by setting a radius from a selected node to study the centrality values in a circular area or by setting the topological radius. This would help us to investigate the manner in which the metrics would change at the local levels. From an emergency perspective, this approach can evaluate the performances of this approach in areas around places of interest such as hospitals or public facilities.

## Supporting information

S1 Dataset Network dataset. (7Z)
